# Why does abortion stigma matter? A scoping review and hybrid analysis of qualitative evidence illustrating the role of stigma in the quality of abortion care

**DOI:** 10.1016/j.socscimed.2022.115271

**Published:** 2022-10

**Authors:** Annik Mahalia Sorhaindo, Antonella Francheska Lavelanet

**Affiliations:** a World Health Organization, Department of Reproductive Health and Research and the UNDP-UNFPA-UNICEF-WHO-World Bank Special Programme of Research, Development and Research Training in Human Reproduction (HRP), 20 Avenue Appia, 1211, Geneva, Switzerland

**Keywords:** Abortion stigma, Qualitative research, Treatment, Thematic analysis, Measurement, Indicators, Quality in abortion care

## Abstract

Abortion stigma shapes the environment in which abortion is delivered and received and can have important implications for quality in abortion care. However, this has not previously been clearly articulated and evidenced. We conducted a scoping review of existing qualitative evidence to characterize the relationship between abortion stigma and quality in abortion care. Using a systematic process, we located 50 qualitative studies to include in our analysis.

We applied the interface of the WHO quality of care and abortion stigma frameworks to the qualitative evidence to capture manifestations of the interaction between abortion stigma and quality in abortion care in the existing literature. Four overarching themes linked to abortion stigma emerged: A) abortion as a sin and other religious views; B) regulation of abortion; C) judgement, labelling and marking; and D) shame, denial, and secrecy. We further characterized the emerging ways in which abortion stigma operates to inhibit quality in abortion care into seven manifestations of the relationship between abortion stigma and quality in abortion care: 1) poor treatment and the repercussions, 2) gatekeeping and obstruction of access, 3) avoiding disclosure, 4) arduous and unnecessary requirements, 5) poor infrastructure and lack of resources, 6) punishment and threats and 7) lack of a designated place for abortion services.

This evidence complements the abortion stigma-adapted WHO quality of care framework suggested by the International Network for the Reduction of Abortion Discrimination and Stigma (inroads) by illustrating specifically how the postulated stigma-related barriers to quality abortion care occur in practice. Further research should assess these manifestations in the quantitative literature and contribute to the development of quality in abortion care indicators that include measures of abortion stigma, and the development of abortion stigma reduction interventions to improve quality in abortion care.

## Highlights

• Studies show a relationship between abortion stigma and quality in abortion care.
• Four abortion stigma themes emerged in our analysis of 50 qualitative studies.
• Analysis combining abortion stigma and quality of care led to seven manifestations.
• Findings were generated from qualitative data from a range of global contexts.
• Future measurement of quality in abortion care should integrate abortion stigma.

## 1 Introduction

Since Goffman's pioneering conceptualization (Goffman, 1963), social science has applied the stigma construct to a range of phenomena. Addressing the limitations of the Goffman definition, Link and Phelan (2001) proposed a reframing of stigma that acknowledges the significance of intersectionality and power; power that is present and exercised within society's institutions (Schaaf et al., 2021), including those that deliver healthcare.

For an array of health conditions, stigma impedes access to high-quality care (Phelan et al., 2015; Knaak et al., 2015; Dell et al., 2021) and is a fundamental cause of health inequalities (Hatzenbuehler et al., 2013). In sexual and reproductive health and rights (SRHR), power, and expressions of power in the form of agenda-setting, for example the dynamics of how SRHR debates are framed, and relationships between abortion seekers and providers have critical implications for health care experiences and outcomes. While commonly present, cohesion around key areas of concern in SRHR, discrimination, and stigma may also manifest differently depending on local forces and contextual dynamics (Schaaf et al., 2021).

When applied to the specific sexual and reproductive health care experience of abortion, stigma has been conceptualized as, a negative attribute that marks individuals, “internally or externally, as inferior to ideals of womanhood” (Kumar et al., 2009) and based on a “… shared understanding that abortion is morally wrong and/or socially unacceptable” (Norris et al., 2011). While equally criticised for its focus on the individual (Millar, 2020), the definition of abortion stigma recognizes the different levels - individual, community, institutional, legal, mass media and cultural - at which the construct operates (Kumar et al., 2009; Hessini, 2014) and how these levels intersect and reinforce one another (Kumar et al., 2009; Hessini, 2014) to shape the environment in which abortion is delivered and received (Seewald et al., 2019; Hussein and Ferguson, 2019; Makleff et al., 2019).

As in other areas of health care, research evidence suggests that abortion stigma experienced or anticipated by both providers and abortion seekers can serve as a barrier to safety and quality in abortion care as defined by the World Health Organization (WHO) (Shellenberg et al., 2011; Juarez and Singh, 2012; Martinez-Hume et al., 2016; Hanschmidt et al., 2016;; Cohen and Joffe, 2021) and has consequences for the psychological and emotional health of abortion seekers (Biggs et al., 2020; Rocca et al., 2020). Stigma is also associated with adverse attitudes towards abortion care policies when measured at the community-level (Cutler et al., 2021) and remains a quality-inhibiting feature of policies and practices in healthcare institutions even in favorable legal contexts (Cárdenas et al., 2018; Cohen and Joffe, 2021). According to the WHO, the delivery of quality health care in health systems should be *effective*, reflect the evidence-base and result in improved health outcomes, based on need; *efficient*, delivered in a way that maximizes use of resources; *accessible*, timely, reasonably located geographically and in a setting where the appropriate skills and resources are available; *acceptable/patient-centred*, taking into account the preferences and desires of service users and their community; *equitable*, without variation according to personal or community characteristics; and *safe*, limiting risks and harm to service users (World Health Organization, 2006). Furthermore, specific to abortion, safety includes consideration of the availability of appropriate services, the legal situation, as well as personal characteristics, such as age and socioeconomic status, as some of the contextual factors that impact the continuum of risk (Ganatra et al., 2014).

Acknowledging the important and pervasive influence that stigma can have on outcomes in health and health care (Keusch et al., 2006; Link and Phelan, 2006), conceptual models have articulated the relationship between abortion legality, stigma and safety (Ostrach, 2016), and previous research has described how interventions may be designed to reduce the impact of stigma in abortion service delivery (Cockrill et al., 2013). However, these do not explicitly describe the ways in which abortion stigma operates to inhibit quality in abortion care.

In 2015, the International Network for the Reduction of Abortion Discrimination and Stigma (inroads) applied an abortion stigma lens to the six dimensions of the WHO quality of care framework. Based on an online forum among providers, activists, academics, and researchers organized by the Network, the WHO framework was modified to outline the stigma-related barriers to quality care and provide a description of the characteristics of “stigma-free” abortion care services (inroads, 2015). For example, restrictive laws and policies were identified as a barrier to *effectiveness*. The separation of abortion care services from other sexual and reproductive health services was considered an *inefficient* use of resources. Shaming of abortion providers into abandoning their practice was described as preventing *access* with recognition that healthcare workers’ negative attitudes towards abortion hamper *acceptability*, while *inequity* is perpetrated by denial of services to marginalized groups. Finally, *safety* was impacted by the lack of routine abortion care training. Although this adaptation of the WHO framework to the context of abortion care commenced a discussion of the ways in which abortion stigma operates to inhibit quality in abortion care, the adaptation was not based on existing evidence, thus limiting its ability to develop targeted interventions aimed at abortion stigma to improve quality in abortion care.

Therefore, we conducted a scoping review of existing qualitative evidence to better inform the relationship between abortion stigma and quality in abortion care. Scoping reviews are useful for assessing the extent, range, and nature of a broad area of research (Arksey and O'Malley, 2005: Levac et al., 2010) and for identifying key characteristics or factors related to a concept (Munn et al., 2018). Qualitative evidence is particularly useful for capturing experiences regarding acceptability, satisfaction, and the overall human experience of engaging with healthcare. An advantage of including qualitative evidence in reviews is that the approach responds to research questions that are not as easily answered using experimental studies (Goldsmith et al., 2007). As we were interested in an aspect of “experience” in abortion care, we agreed that qualitative evidence was best suited to answer our questions and decided against including quantitative evidence in this review (Dixon-Woods et al., 2006; Goldsmith et al., 2007; Thomas and Harden, 2008).

We set out to answer the following questions:

● What manifestations of abortion stigma are associated with quality in abortion care?
● How do manifestations of abortion stigma potentially impact upon quality in abortion care?

This scoping review identified various manifestations of the way in which abortion stigma and quality in abortion care are interrelated. Such information can inform interventions aimed at addressing abortion stigma to improve quality in abortion care.

## 2 Methodology

We initially conducted a search of four bibliographic databases: Web of Science, CINAHL, PubMed, Popline, to identify all articles published through to June 1, 2018. Although we customized the strategies based on the electronic database searched, all search strategies combined two main concepts: abortion stigma and quality of care and were limited to retrieve studies including a qualitative component. We conducted an updated search through to September 30, 2020 using an abbreviated search strategy. While this approach may not capture all available evidence, we used databases that previously provided the greatest retrievals and tested the abbreviated search strategy on the prior timeframe to ensure similar results. The complete search strategy can be found in Appendix 1 to this manuscript. We report this review according to PRISMA (Preferred Reporting Items for Systematic Reviews and Meta-Analyses) guidelines (Moher et al., 2015) (Appendix 2).

We included studies if they were a) published in English or Spanish, b) included qualitative evidence of abortion stigma at the level of the individual, community, institutions, legislation, or mass media and culture; c) and if they reported elements that could be linked to the direct influence of stigma in quality in abortion care. We excluded studies that did not discuss a manifestation of abortion stigma as it related to quality in abortion care, did not explain the study methodology, analysis or process of data collection, or did not report directly on primary or secondary data analysis.

Two reviewers (AMS, AFL) independently conducted the screening, full text review, and data extraction. Titles and abstracts were screened using the Covidence tool (Covidence systematic review software, Veritas Health Innovation, Melbourne, Australia. Available at www.covidence.org); full texts were obtained for studies where both reviewers deemed them eligible. We used a standardized extraction form to collect information about each study including study setting, study type and methodology, participant characteristics, and relevant stigma and quality of care thematic findings.

We used a hybrid approach (Swain, 2018) to this qualitative analysis to incorporate both a deductive theoretical process, informed by the interface of two existing frameworks (abortion stigma and WHO quality of care) and an inductive, data-driven approach to identify themes and manifestations. The hybrid approach enabled us to build on the existing inroads framework while generating data and new defining information on this phenomenon from the literature (Swain, 2018). This approach required an iterative process whereby we discussed, extracted, organized and reorganized excerpts from the literature until we reached consensus. We did not conduct an appraisal of included studies in line with scoping review methodology (Munn et al., 2018). Finally, there is no registered protocol for this review.

We present the analysis using a narrative synthesis (Creswell, 2007), where the results are organized thematically and described, and supplemented with tables of descriptive characteristics (Table 1).

**Table 1:** *Characteristics of included studies*.

	Author, year	Country	Sample size and study participants	Main study aim	Date collection method
1	Aniteye and Mayhew, 2013	Ghana	43 Health professionals: 15 Ob/Gyns^a^ 14 Midwives 7 Pharmacists 7 Other health professionals	To investigate abortion policy implementation	In-depth interviews
2	Cárdenas et al., 2018	Uruguay	20 participants: 10 abortion clients aged 22-38 10 health professionals including physicians, midwives, social workers, and a psychiatrist	To analyze opinions and attitudes of both abortion clients and health professionals following decriminalization and assesses how abortion stigma manifests	In-depth interviews
3	Cleeve et al., 2017	Uganda	17 women between the ages of 15 and 24 years	To explore reproductive agency in relation to unsafe abortion among young women seeking post-abortion care	In-depth interviews
4	Dahlback et al., 2007	Zambia	34 adolescent girls selected from a larger study population	To describe the situation of adolescent girls admitted to the hospital after having resorted to unsafe induced abortion	In-depth interviews
5	Deb et al., 2020	Australia	25 General Practitioners from 24 practices	To describe GP medical abortion delivery models	In-depth interviews
6	de Vries et al., 2020	Benin, Cameroon, Côte d'Ivoire, Kenya, Mali, Mozambique, Panama, Peru, Uganda, and Zambia	127 participants: Ob/Gyn professional society members 12 other professional body representatives 21 policy officers 38 NGO and multilateral organization representatives 15 others	To report on the cross-country analysis of legal, political, sociocultural, and professional contexts that ObGyn societies work in and to reflect on the capabilities, barriers, opportunities, and identified strategies to strengthen their role in safe abortion advocacy	In-depth interviews
7	Doran and Hornibrook, 2014	Australia (new South Wales)	13 women	To identify factors that impact the experience of rural women in accessing abortions	In-depth interviews
8	Esia-Donkoh et al., 2015	Ghana	21 young people (aged 12 to 24) who had their abortion three months prior to the study	To examine the pre and post abortion experiences among young females	In-depth interviews
9	Fathallah (2019)	Lebanon	119 participants: 84 women who had obtained an abortion (ages 18–65) 35 physicians who offer abortion services	To explore the intersectional effects of criminalization on women's access to safe abortion	In-depth interviews
10	Favier et al., 2018	South Africa	9 participants: 4 medical practitioners 1 government official 2 NGO staff 2 other	To examine the country's approach to the implementation of a national abortion service program, after a change in law or policy guideline	In-depth interviews
11	Fernandez Vázquez, 2019	Argentina	27 health providers: 16 general practitioners 4 gynaecologists 2 social workers 2 psychologists 1 sociologist 1 paediatrician 1 pharmacist	To understand abortion policies in Argentina between 2007 and 2017	In-depth interviews
12	Fielding et al., 2002^b^	United States (New York)	30 of 43 women following their abortion	To gain insights into how patients view induced abortion using mifepristone	In-depth interviews
13	Freedman et al., 2010	United States	30 OBGYNs	To explore the professional barriers that recent graduates of OB/GYN residency programs face when they wish to provide abortions	In-depth interviews
14	Freeman and Coast, 2019	Zambia	51 participants: 3 clinical officers 8 community health workers 3 district medical officers 6 doctors (non-specialist) 12 midwives 5 nurses 14 OB/GYNs	To consider the experiences of practitioners who conscientiously object to abortion alongside those who do not in order to investigate divergences – or similarities	In-depth interviews
15	Harden and Ogden, 1999	United Kingdom	54 young women aged between 16 and 24 following their abortion	To describe women's experiences of arranging and having an abortion	In-depth interviews
16	Heller et al., 2016	United Kingdom (Scotland)	16 women	To explore the experiences, including encountered barriers, of women from a remote and rural setting who had a termination of pregnancy	In-depth interviews
17	Homaifar et al., 2017	United States (Nebraska)	431 of 496 clinicians	To understand the motivations around and practices of abortion referral among women's health providers	Survey
18	Hulme-Chambers et al., 2018	Australia (Victoria)	18 women aged 16 years and over	To explore which aspects of a rural medical termination of pregnancy service system worked well, and what could be improved	In-depth interviews
19	Ireland et al., 2020	Australia	11 women who had experienced a telemedicine abortion within the last 6 months	To explore and better understand women's access to telemedicine abortion in Australian rural areas	In-depth interviews
20	Izugbara et al., 2015	Kenya	50 women treated for complications of unsafe abortion selected from a larger study population	To address the knowledge gap regarding the social dimensions of abortion safety	In-depth interviews
21	Izugbara et al., 2017	Kenya	35 post-abortion care providers; 51 interviews, 10 focus groups	To explore abortion care providers' constructions of the challenges that unmarried young women and girls face in relation to abortion care-seeking	In-depth interviews; Focus Group Discussions
22	Jayaweera et al., 2018	Kenya	71 women and girls aged 15 to 35; 7 focus groups	To gain knowledge about women's experiences seeking and accessing abortion in informal settlements	Secondary analysis of focus group discussion data
23	Kavanaugh et al., 2019	United States (Michigan, New Mexico)	29 women seeking abortion services	To assess regarding information barriers that individuals may encounter and strategies for circumventing these barriers	In-depth interviews
24	Kimport et al., 2012	United States	41 women over 18 years old participating in two studies, who had received an abortion or were planning an abortion	To describe some of the ways lived experience may reinforce or counter the social myths about abortion clinics	In-depth interviews
25	Kimport et al., 2016	United States	61 Ob/Gyns participating in two studies	To investigate whether the current abortion provision landscape shares the pre-Roe interpersonal patterns of physicians choosing which abortions to perform or coordinate care for based on social criteria rather than medical ones	In-depth interviews
26	LaRoche and Foster, 2018	Canada	41 women with a total of 87 abortions in the 5 years preceding the interviews	To understand better the ways that women who have had multiple abortions talk about and view those experiences	In-depth interviews
27	LaRoche et al., 2020	Australia	22 women, transgender folks, and gender non-binary individuals who had used mifepristone for abortion	To explore the experiences of abortion patients obtaining mifepristone through different service delivery models in different geographic areas	In-depth interviews
28	Leyandowski et al., 2012	Malawi	485 Malawian policymakers, governmental employees, educators, healthcare providers, religious leaders, nongovernmental organization members, and community members	To investigate community-level opinions on the social consequences of unwanted pregnancy and unsafe abortion	In-depth interviews
29	Linton, 2020	United States	37 health practitioners: 33 physicians 4 advanced practice clinicians	To describe current abortion referral patterns among generalist obstetrician gynecologists and primary care practitioners	In-depth interviews
30	Loganathan et al., 2020	Malaysia	44 individuals (37 interviews) representing: 13 medical doctors 10 civil society organizations 5 industry 4 migrant workers 4 international organizations 3 trade union 3 academia 2 other policy stakeholders	To explore policy and the provision of sexual and reproductive health services for migrant workers in Malaysia	In-depth interviews
31	Makleff et al., 2019	Kenya, India	45 participants: 24 in Kenya 21 in India (2 focus groups with 11 participants each)	To examine the experiences of women who obtained an abortion with regard to stigma, expectations, and perceptions of abortion quality of care.	In-depth interviews; Focus Group Discussions
32	Margo et al., 2016	United States (South Carolina)	45 women aged 18 or older seeking abortion care	To explore how women sought information, communicated with professionals, received referrals (or did not) and prepared for their abortion appointments	In-depth interviews
33	Marlow et al., 2014	Kenya	10 focus groups with 8–14 participants each, split into married women aged 24–49 and unmarried women 20 years or younger	To understand the different methods used, including which providers were utilized in the community,	Focus group discussions
34	McLean et al., 2019	Ethiopia	29 providers 3 focus groups (2 groups of 5; 1 group of 3)	To explore abortion service providers' reflections of their work, their perceptions and interpretations of the abortion law, and the potential ethically challenging aspects of their work	In-depth interviews; Focus Group Discussions
35	Mohamed et al., 2018	Kenya	12 young women (18–24 years) who received induced or post-abortion services that day	To characterise the quality, barriers, cultural beliefs and community norms around induced abortion and post- abortion seeking	In-depth interviews
36	Nandagiri, 2019^,^^b^	India	21 or 188 survey participants	To explore community health intermediaries' attitudes and explanations of roles in and knowledge of abortion	In-depth interviews
37	Påfs et al., 2020	Rwanda	52 participants: 32 interviews, 5 focus groups (4–6 participants in each)	To explore how health care providers understand the amended law and implement it into their clinical practice	In-depth interviews; Focus Group Discussions
38	Palomino et al., 2011	Peru	52 participants; 19 interviews, 4 focus groups	To improve understanding of how men and women make reproductive decisions	In-depth interviews; Focus Group Discussions
39	Payne et al., 2013	Ghana	4 physicians	To better understand the reproductive health and abortion services	In-depth interviews
40	Pheterson and Azize, 2008	St. Martin, St. Maarten, Anguilla, Antigua and St Kitts	26 physicians: 12 OB/GYNs 11 family practitioners 3 physician government administrators	To examine abortion practices in juridically separate health systems as one service network	In-depth interviews
41	Puri et al., 2012	Nepal	35 health care workers: 14 OB/GYNs 13 nurses 6 administrators 1 health assistant 1 counselor	To examine health care workers' views of abortion legalization, and changes that they have observed in their practices	In-depth interviews
42	Raifman et al., 2018	Tunisia	23 participants: 7 physicians 10 midwives 2 nurses 4 gatekeepers	To explore provider beliefs about abortion, abortion safety and legality, and contraception, and whether these beliefs correspond to their actions with respect to abortion counseling, provision, denial, and referral	In-depth interviews
43	Schwandt et al., 2013	Ghana	122 participants 50 post-abortion clients; 20 interviews, 4 focus groups 32 male partners; 19 interviews 2 focus groups 17 family planning nurses; 11 interviews, 1 focus groups 23 OB/GYNs; 8 interviews, 2 focus groups	To understand the decision-making process associated with induced abortion	In-depth interviews; Focus Group Discussions
44	Seewald et al., 2019	North America, South America and Africa	3 stories	To explore the possible relationships between stigma and abortion complications, considering stigma experienced by patients and healthcare provider	Reflection workshops
45	Sri and Ravindran, 2012	India	14 focus groups with 12–15 women in each	To understand how rural and other groups of marginalized women define safe abortion; their perspectives and concerns regarding medical abortion (MA); and what factors affect their access to safe abortion	Focus group discussions
46	Suh (2018)	Senegal	89 participants (including health workers MOH officials; personnel from national and international NGOs and research agencies; law enforcement officials; members of legal and medical professional associations; feminist advocates; parliamentarians; and journalists	None provided	In-depth interviews
47	Teffo, 2017^b^	South Africa	30 termination of pregnancy providers	To determine the proportion of designated termination of pregnancy (TOP) facilities in the public sector that actually provide these services; explore the factors that influence the provision of TOP services; and explore the work experiences of TOP providers at designated facilities	In-depth interviews
48	Ushie et al., 2019	Kenya	72 interviews, 18 focus group discussions	To understand community-level perceptions of abortion and to explore access and use of abortifacient pharmaceutical drug	In-depth interviews, Focus group discussions
49	Weitz and Cockrill, 2010	United States	20 abortion patients	To explore abortion clinic patients' opinions about receiving abortions from general women's health care providers	In-depth interviews
50	Yegon et al., 2016	Kenya	12 focus groups in Eastern region 14 focus groups in Rift valley region	To explore abortion-related stigma at the community level as a barrier to women realizing their rights to a safe, legal abortion and compare manifestations of abortion stigma	Focus group discussions

a Ob/Gyns = Obstetrician Gynaecologists.

b Represent studies with mixed method design.

## 3 Results

### 3.1 Locating studies on abortion stigma and quality in abortion care

**Fig. 1:**
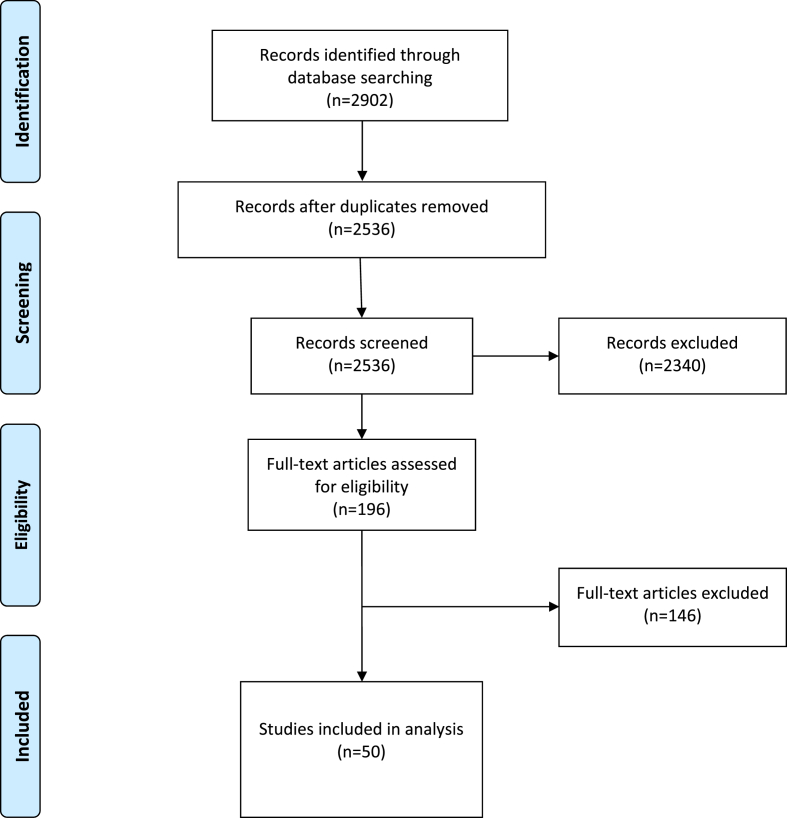
PRISMA Flow Diagram of included studies.

We summarize the process undertaken to select sources for this review in the PRISMA below (Fig. 1). Our database search produced 2536 titles and abstracts once duplicates were removed. Following the screening process described above, 2340 titles and abstracts were excluded, and 196 full texts were retrieved and assessed for eligibility according to the criteria outlined above. Of these, 144 were excluded and 50 were included in the analysis.

### 3.2 The intersection of abortion stigma and abortion quality care

To determine which aspects of abortion stigma were emerging in our evidence, we first investigated occurrences of abortion stigma in our data and identified four recurring themes: A) abortion as a sin and other religious views; B) regulation of abortion; C) judgement, labelling and marking; and D) shame, denial, and secrecy. The definitions for each of these themes are described in Table 2Table 2.

**Table 2:** *Definitions of themes*.

Abortion stigma	
Theme A. Abortion as a sin and other religious views	A religious sin that offends some spiritual principles
Theme B. Regulation of abortion	Sanction of abortion under certain circumstances over others
Theme C. Judgement, labelling and marking	Identified as engaging in deviant behavior and therefore, holding a negative perception of a person for seeking or providing abortion
Theme D. Shame, denial, and secrecy	Humiliation or distress related to involvement in abortion leading to concealing that one has had an abortion or is involved in abortion care
Manifestations of abortion stigma on quality in abortion care	
Theme 1. Poor treatment and the consequences	Negative engagement with providers and health care personnel when seeking abortion services Perceived or real consequences associated with accessing abortion Resorting to clandestine abortion
Theme 2. Gatekeepers/obstructing access	Not providing women with information/tools/resources/referral to access abortion or discouraging women from requesting services Preventing or limiting training and access to equipment
Theme 3. Tactics for avoiding disclosure	Actions taken to prevent abortion from becoming known
Theme 4. Arduous/unnecessary requirements	Unnecessary delays, tests or treatments, unreasonable costs
Theme 5. Poor infrastructure and lack of resources	Failure to fully implement standards and protocols Perceived or real consequences associated with performing abortion
Theme 6. Punishment/threats	Penalty or retribution inflicted on a person or persons as a result of their involvement in abortion care
Theme 7. No designated place for abortion services	Lack of defined areas for women seeking abortion services,

These overarching themes in abortion stigma intersected with quality in abortion care as described in the seven manifestations described below: 1) poor treatment and the repercussions, 2) gatekeeping and obstruction of access, 3) avoiding disclosure, 4) arduous and unnecessary requirements, 5) poor infrastructure and lack of resources, 6) punishment and threats and 7) lack of a designated place for abortion services. Evidence from individual studies frequently contributed to more than one manifestation. Furthermore, the seven themes are interrelated and overlap. As such, the below descriptions include some repetition. Finally, in discussing this evidence, we attempt to use gender-inclusive language where possible.

1. Poor treatment and implications for quality

Thirty-one studies described the poor treatment of abortion seekers and how such treatment can lead people to resort to clandestine or unsafe abortion (Cárdenas et al., 2018; de Vries et al., 2020; Esia-Donkoh et al., 2015; Favier et al., 2018; Fielding et al., 2002; Freedman et al., 2010; Harden and Ogden, 1999; Izugbara et al., 2015, 2017; Jayaweera et al., 2018; Kimport et al., 2012; LaRoche and Foster, 2018; LaRoche et al., 2020; Leyandowski et al., 2012; Loganathan et al., 2020; Makleff et al., 2019; Margo et al., 2016; Marlow et al., 2014; McLean et al., 2019; Mohamed et al., 2018; Nandagiri, 2019; Påfs et al., 2020; Palomino et al., 2011; Payne et al., 2013; Pheterson and Azize, 2008; Puri et al., 2012; Schwandt et al., 2013; Sri and Ravindran, 2012; Suh, 2018; Ushie et al., 2019; Weitz and Cockrill, 2010). Abortion seekers described being subjected to judgment and insensitivity from unsupportive providers (Cárdenas et al., 2018; Favier et al., 2018; Fielding et al., 2002; Harden and Ogden, 1999; Izugbara et al., 2017; Makleff et al., 2019; Margo et al., 2016; Marlow et al., 2014; McLean et al., 2019; Mohamed et al., 2018; Palomino et al., 2011; Puri et al., 2012; Suh, 2018). In the literature, people seeking abortions explained that many providers framed abortion as bad, harmful, sinful, or as killing of “a life force” (Nandagiri, 2019), and behaved hostile, moralistic and cold because they [the pregnant person] “should know better … [than] to get pregnant” (Fielding et al., 2002). Some abortion seekers reported being insulted, where doctors spoke ill of them (Marlow et al., 2014), or facing health workers who were “unsympathetic, disrespectful, [and] rude” (Favier et al., 2018), which ultimately led the pregnant person to deter seeking care. Abortion seekers also described feeling as though health providers viewed them as irresponsible (Harden and Ogden, 1999) and, as a result, were provided with no emotional support (Kimport et al., 2012).

Judgment manifested in several different ways. One study included the inappropriate questioning or interrogation of abortion seekers' choice to terminate their pregnancies by healthcare workers (Harden and Ogden, 1999), second-guessing the person's decision and attempting to persuade them to reconsider. In another study, people reported that hospital-based providers called them names and breached confidentiality by publicizing their abortion (Izugbara et al., 2015). Judgment also existed across a variety of settings. Abortion seekers found pharmacists to be judgmental in their behaviors (exhibited through rude gestures) towards them when filling prescriptions related to abortion medications (Hulme-Chambers et al., 2018). Judgmental reactions from providers led abortion seekers to be secretive about their abortion to protect themselves (Margo et al., 2016) or refuse to see their known physicians for fear of judgment (Påfs et al., 2020).

Health care providers were reported as having a lack of empathy and exhibiting insensitivity toward abortion seekers (Aniteye and Mayhew, 2013; Harden and Ogden, 1999), which had implications for abortion seekers' experience and for their care. In a study focused on young people, the authors described how, when attending facilities seeking care for complications, they were treated “discourteously”, scolded (Izugbara et al., 2017) and left alone to suffer shame, as providers shunned them. One article suggested that treatment varied depending upon abortion seekers’ expressed remorse or regret for the unplanned pregnancy or the abortion (Kimport et al., 2016). Awareness of the poor treatment of other people often discouraged those seeking abortion from attending facilities.

Abortion seekers also spoke of the impersonality of some clinics, as they felt they were “herded [] in like cattle” (Fielding et al., 2002). Where people were separated from their companions, the experience felt isolating and lonely (Kimport et al., 2012). Even interactions with abortion protestors at the health care center were seen to be further stigmatizing (Doran and Hornibrook, 2014; Kimport et al., 2012). For example, one participant explained how “strangers [were] waving placards, telling [them] what to do with [their] body” (Doran and Hornibrook, 2014). Where there were protesters outside the building, some abortion seekers took this to mean that the providers in the facility “… did not care about protecting patients like [them] …” (Kimport et al., 2012). Although providers often take measures to eliminate or limit such protester presence near facilities, they have little control over this aspect of people's experience.

The experience or fear of poor treatment often led people to seek unsafe abortion (Freedman et al., 2010; Jayaweera et al., 2018; Leyandowski et al., 2012; Makleff et al., 2019; Payne et al., 2013) including from traditional healers (Loganathan et al., 2020). Some people preferred to seek services outside of the facility where access to services was more like a business interaction because “when [you] go to the backstreet abortionist, they don't ask you questions. You come and you say, ‘I want this,’ they sell you the drug” (Favier et al., 2018). The use of informal or non-facility-based services also meant that abortion seekers could protect their anonymity. In one study, a participant described the risks of seeking care at a high-profile health facility “because they will keep your file, and everybody will know what you came to do” (Izugbara et al., 2015). Physicians also noted risks to privacy because “people are labelled, [and] anyone who goes to sit on [one particular] bench outside in a very busy part of the hospital” is known to be presenting for an abortion (Payne et al., 2013). In some locations, people seeking abortion services are not prioritized. Providers tend to other services before caring for those seeking abortion (Schwandt et al., 2013).

2. Gatekeepers of abortion obstructing access

Twenty-six studies (Aniteye and Mayhew, 2013; Cárdenas et al., 2018; de Vries et al., 2020; Doran and Hornibrook, 2014; Esia-Donkoh et al., 2015; Fathallah, 2019; Freedman et al., 2010; Freeman and Coast, 2019; Harden and Ogden, 1999; Heller et al., 2016; Homaifar et al., 2017; Hulme-Chambers et al., 2018; Izugbara et al., 2015; Izugbara et al., 2017; Kimport et al., 2016; LaRoche et al., 2020; Margo et al., 2016; Nandagiri, 2019; Marlow et al., 2014; Påfs et al., 2020; Payne et al., 2013; Pheterson and Azize, 2008; Raifman et al., 2018; Schwandt et al., 2013; Ushie et al., 2019; Weitz and Cockrill, 2010) described instances of providers acting as gatekeepers to abortion care, including actions taken to actively discourage abortion seekers from accessing services. About one-third of these studies (de Vries et al., 2020; Fathallah, 2019; Freeman and Coast, 2019; Freedman et al., 2010; Heller et al., 2016; Homaifar et al., 2017; Hulme-Chambers et al., 2018; Kimport et al., 2016; Margo et al., 2016; Pheterson and Azize, 2008) focused on matters directly related to the delivery of timely services or issues related to geography (i.e. accessibility). Participants in these studies experienced significant delays due to encounters with providers unwilling to provide services or referrals. These delays were often compounded by the experience of additional legal or regulatory barriers, including the need to obtain an ultrasound, medications from a specific pharmacy (Hulme-Chambers et al., 2018) or additional authorizations (de Vries et al., 2020; Freedman et al., 2010).

In a few studies, participants specifically described their providers’ obstructionist behavior. Providers intentionally misled abortion seekers by providing false referrals to adoption agencies, crisis pregnancy centers or therapists (Homaifar et al., 2017; Margo et al., 2016). In some of these cases, providers felt that abortion seekers needed additional counseling, aware that crisis pregnancy centers, specifically, would dissuade individuals from having an abortion (Homaifar et al., 2017). Abortion seekers in one study viewed requirements for unnecessary testing and multiple visits as an attempt to deter care (Heller et al., 2016). Linked to instances of judgment discussed in the section above, providers also actively tried to dissuade individuals in their consultations (Aniteye and Mayhew, 2013) “encourag[ing] [] mother[s] not to end the life” of their infants (de Vries et al., 2020; Freeman and Coast, 2019; Homaifar et al., 2017). Providers justified their obstruction based on beliefs that abortions lead to ill-health (Nandagiri, 2019) or prolonged “weakness” impacting fertility (Nandagiri, 2019).

Regardless of the legal framework, providers often used discretionary measures to determine which abortion seekers were deserving of services, raising questions about equitability of access. Factors such as marital status, presence of existing children, age, educational attainment, and acceptance of contraception were preconditions to the receipt of services (Fathallah, 2019; Freeman and Coast, 2019). Providers made distinctions between elective and indicated procedures (Freeman and Coast, 2019; Pheterson and Azize, 2008), and consideration was given to whether the reasons were a “genuine problem” (Aniteye and Mayhew, 2013) or “convincing enough” to prove that they really needed the abortion (Freeman and Coast, 2019). Some studies spoke to issues of disproportionate impact among certain groups, including students (Marlow et al., 2014) and people with several children (Pheterson and Azize, 2008). For adolescents specifically, shame was exacerbated (Marlow et al., 2014), especially where they received care alongside adults (Izugbara et al., 2017), or if they were unmarried (Payne et al., 2013; Suh, 2018). Providers were also more comfortable offering counseling. In settings where abortion committees operated as a screening and approval mechanism, abortions that were not medically indicated and were considered “elective” were not granted approval (Freedman et al., 2010; Kimport et al., 2016).

Gatekeeping not only occurred at the level of patient interaction with providers, but also at the institutional level related to hiring providers or offering services in a particular facility. In one study, individual providers described the concessions made upon accepting new employment whereby they agreed to not perform abortions (Freedman et al., 2010). In another study, junior doctors were discouraged from providing and even discussing abortion care (Freeman and Coast, 2019). In some cases, this led to system inefficiencies, as providers, including those with abortion training, were unable or unwilling to provide services (Izugbara et al., 2017; Teffo and Rispel, 2017). Restrictions in practice extended to referrals in some cases, where healthcare professionals were instructed by colleagues not to make referrals (Freedman et al., 2010). Private practices were also restricted from providing services by building owners with anti-choice views (Freedman et al., 2010) or by institutions that were religiously affiliated (Freedman et al., 2010; Kimport et al., 2016). In two studies, departure from public institutions to private facilities arose as an opportunity to apply additional charges (Freedman et al., 2010). Informal fees often led abortion seekers to resort to unsafe services (de Vries et al., 2020; Loganathan et al., 2020; Ushie et al., 2019).

Eight of the 26 studies spoke to issues linking gatekeeping and user-centred care (Doran and Hornibrook, 2014; Esia-Donkoh et al., 2015; Harden and Ogden, 1999; Hulme-Chambers et al., 2018; LaRoche et al., 2020; Påfs et al., 2020; Raifman et al., 2018; Weitz and Cockrill, 2010). Participants described the unacceptable nature of facing providers who were reluctant or refused to provide care (Doran and Hornibrook, 2014; Påfs et al., 2020; Raifman et al., 2018; Weitz and Cockrill, 2010). Even where participants expressed clear desires to proceed with an abortion, providers presented an obstacle to accessing needed information and care (Harden and Ogden, 1999; Hulme-Chambers et al., 2018; Påfs et al., 2020; Raifman et al., 2018). Instead, participants desired staff that were friendly, compassionate, and sympathetic to their needs and individual circumstances (Esia-Donkoh et al., 2015; LaRoche et al., 2020; Raifman et al., 2018).

Five studies (Cárdenas et al., 2018; Izugbara et al., 2015, 2017; Payne et al., 2013; Ushie et al., 2019) spoke to the relationship between gatekeeping and safety. Some providers perceived themselves to be in a difficult situation as they did not want to perform abortions, but they wanted to prevent death, describing scenarios of abortion seekers presenting “when they are almost dead … in very bad condition” (Izugbara et al., 2017). However, in some cases, gatekeepers themselves created safety issues by turning away abortion seekers and refusing to make necessary referrals (Payne et al., 2013). Furthermore, lack of access to services due to cost emerged as an issue (Payne et al., 2013), as only post abortion care is routinely covered by insurance. Participants viewed unaffordability, something they saw to be controlled by various gatekeepers, as something that was directly linked to safety (Izugbara et al., 2015; Ushie et al., 2019). Some described interactions with pharmacists, who deliberately “hike the price because you are desperate”, and who will only sell abortifacients to those known to the pharmacy (Ushie et al., 2019). Others spoke of places that will “make you pay heavily even when you say you don't have money” (Izugbara et al., 2015); in both cases, safer options became inaccessible due to cost.

Structural and institutional abortion stigma also manifested as an extension of the implications of conscientious objection in one study (Freeman and Coast, 2019). Similar to other instances of provider gatekeeping described above, in health care teams, the views of senior physicians influenced the behavior of junior members of their team, as the senior doctors actively dissuaded these physicians from becoming involved in any capacity in abortion care. In some cases, this included risks to career advancement where junior providers chose to participate in such care (Freeman and Coast, 2019).

3. Tactics for avoiding disclosure related to abortion

The failure to acknowledge existing abortion services, as well as the tactics used to do so were discussed in 22 studies (Aniteye and Mayhew, 2013; Cárdenas et al., 2018; Dahlback et al., 2007; Deb et al., 2020; de Vries et al., 2020; Esia-Donkoh et al., 2015; Fernández Vázquez and Brown, 2019; Freedman et al., 2010; Homaifar et al., 2017; Ireland et al., 2020; 20, Margo et al., 2016; Påfs et al., 2020; Payne et al., 2013; Pheterson and Azize, 2008; Puri et al., 2012; Raifman et al., 2018; Sri and Ravindran, 2012; Suh, 2018; Ushie et al., 2019; Weitz and Cockrill, 2010; Yegon et al., 2016). Providers did not advertise their services due to stigma and fear of legal repercussions (Aniteye and Mayhew, 2013; Deb et al., 2020; de Vries et al., 2020; Seewald et al., 2019) (also see manifestation 6, Punishment and threats). Providers did not want to be known as abortion providers among colleagues, as well as within their communities (Aniteye and Mayhew, 2013; Freedman et al., 2010; Homaifar et al., 2017). Abortion seekers also avoided disclosure of their desired abortions with their regular providers, especially when they shared community networks, for fear of disrupting their relationships (Esia-Donkoh et al., 2015; Ireland et al., 2020; Margo et al., 2016; Påfs et al., 2020; Puri et al., 2012; Sri and Ravindran, 2012). Some studies described the use of private physicians by migrants or those in rural villages as a deliberate attempt to avoid disclosure (Pheterson and Azize, 2008; Sri and Ravindran, 2012), while others described care seeking behavior based on the reputation of a provider to maintain confidentiality and privacy (Izugbara et al., 2015).

Misclassification of abortions in medical registries was another tactic for avoiding disclosure. Abortion procedures were described as spontaneous abortions, as “hemorrhagic management” or management of “incomplete” abortion (Suh, 2018). Other examples of misclassification of diagnosis included “diagnostic D&C” or “preeclampsia” (de Vries et al., 2020). Some providers described the use of “medical records that were not official” or the use of aliases on prescriptions to ensure secrecy (Fernández Vázquez and Brown, 2019).

While these studies speak to the various components of quality care, links to safety specifically emerged in 8 of the 22 studies (Cárdenas et al., 2018; Dahlback et al., 2007; Freedman et al., 2010; Påfs et al., 2020; Pheterson and Azize, 2008; Puri et al., 2012; Sri and Ravindran, 2012; Ushie et al., 2019). The desire to avoid disclosure led abortion seekers to self-manage, seek clandestine services, or seek care from traditional healers (Cárdenas et al., 2018; Cleeve et al., 2017; Freedman et al., 2010; Påfs et al., 2020; Suh, 2018; Ushie et al., 2019); in some cases, this secrecy extended to the parents of abortion seekers who assisted in unsafe practices (Suh, 2018). In one study, providers working in a context where abortion was illegal noted that secrecy ensured safety; individuals had access to safe abortions but pushing legalization and exposing providers would lead to an uprising in conservative groups compromising access to existing safe services (Pheterson and Azize, 2008).

4. Arduous/unnecessary requirements

Six studies described the often arduous and unnecessary steps abortion seekers were required to take to receive services (Cárdenas et al., 2018; de Vries et al., 2020; Fathallah, 2019; Freeman and Coast, 2019; Nandagiri, 2019; Raifman et al., 2018) because of negative attitudes towards abortion or systems and institutions that stigmatized abortion services. In one study (Cárdenas et al., 2018), abortion seekers described the need to take a five-day reflection period before they were permitted to receive abortion services. The study participants described this requirement as “excessive, unnecessary and torturous” especially because they were already confident in their decision. Rather, they felt that delaying the procedure was disrespectful and in fact was designed to stigmatize and challenge their decisions to terminate the pregnancy. In this study (Cárdenas et al., 2018), providers themselves felt this imposed stipulation could possibly do harm. In another study (Freeman and Coast, 2019), providers who were able to conduct the procedure but merely preferred not to, referred elsewhere, which also contributed to unnecessary delays (Freeman and Coast, 2019).

Many of these were required by law or were institutional policies. In one study (de Vries et al., 2020), a national multidisciplinary committee was required to approve therapeutic abortions, all of which were documented in a national registry. In another study, providers described requiring consent from other family members, before agreeing to perform the procedure (Nandagiri, 2019). Abortion seekers were also refused services if they did not have the required paperwork, such as a letter, marriage contract or identification card (Raifman et al., 2018), or if they could not adequately convince providers of their need for the procedure during counseling (Fathallah, 2019; Freeman and Coast, 2019).

5. Poor infrastructure and lack of resources

Fourteen studies described challenging working conditions for providers primarily due to poor infrastructure or lack of resources (Cárdenas et al., 2018; de Vries et al., 2020; Freeman and Coast, 2019; Hulme-Chambers et al., 2018; Izugbara et al., 2017; Kavanaugh et al., 2019; Linton et al., 2020; Margo et al., 2016; Nandagiri, 2019, Påfs et al., 2020; Payne et al., 2013; Raifman et al., 2018; Schwandt et al., 2013; Teffo and Rispel, 2017). Having access to information about where safe abortion services exist or how to make appropriate referrals was identified as a problem in several studies. Specifically, providers lacked information about where to direct people seeking safe services (de Vries et al., 2020; Freeman and Coast, 2019; Hulme-Chambers et al., 2018; Kavanaugh et al., 2019; Linton et al., 2020; Nandagiri, 2019); with one further study identifying the need for feedback mechanisms between health institutions once referrals were made (Freeman and Coast, 2019). Some providers expressed frustration due to their inability to tap into a network to share cases publicly, which meant that they could not “solve problems on a wider scale” and benefit from collective learning (Schwandt et al., 2013). Personal safety concerns linked to abortion provision were also raised in one study, with participants noting that abortion opponents ‘‘can break down the house” (Schwandt et al., 2013).

As suggested above, abortion providers also received little support from program and facility managers, as abortion services were not seen as a priority in system planning (Cárdenas et al., 2018; Teffo and Rispel, 2017), leaving providers to “play a dual role as both the provider and the manager” and work “within a structure that is not well equipped” (Teffo and Rispel, 2017). Where services were offered, they were often arranged in such a way that led to delays, either due to rotating schedules of the very few providers available (Freeman and Coast, 2019; Margo et al., 2016) or due to overcrowded facilities (Payne et al., 2013) and limited space (Raifman et al., 2018). Medical abortion commodities (Raifman et al., 2018), as well as equipment (Izugbara et al., 2017; Schwandt et al., 2013) such as ultrasound and forceps, including for second trimester care provision (Payne et al., 2013), were described as inadequate. Lack of training was also an issue (Cárdenas et al., 2018; Påfs et al., 2020; Payne et al., 2013; Raifman et al., 2018).

6. Punishment and threats

Evidence from seven studies suggested that abortion seekers and providers are threatened and punished in diverse ways when looking to terminate a pregnancy (Freedman et al., 2010; Makleff et al., 2019; Påfs et al., 2020; Payne et al., 2013; Pheterson and Azize, 2008; Seewald et al., 2019; Ushie et al., 2019). These behaviors and practices are often linked to opposition to and negatives concepts of abortion.

Abortion seekers and providers often feared criminal liability for being involved with the delivery or receipt of abortion services. In some countries, abortion seekers feared that health care workers would report them to the authorities for seeking abortion (Makleff et al., 2019). In one study, a health care worker described having experienced individuals in their facilities being handcuffed and questioned about whether the abortion they were experiencing was spontaneous or induced (Påfs et al., 2020).

Fear also impacted providers as well, as they were often deterred or prevented from offering services from leadership in their health care institutions, as alluded to above (Freedman et al., 2010; Påfs et al., 2020). For those that provided abortions, they saw their care as a calculated risk relying on the fact that few cases were tested legally (Payne et al., 2013), or only providing services to known individuals (Ushie et al., 2019). As mentioned in previous sections, providers, including pharmacists, feared legal consequences including fines or imprisonment (Påfs et al., 2020; Payne et al., 2013; Seewald et al., 2019; Ushie et al., 2019). These fears were magnified for some providers who were suspicious of government motives, believing that covert provision was encouraged as “an institutionalized toleration system” (Pheterson and Azize, 2008). Specifically, one provider stated that “[e]veryone knows [abortions] are done” and the “Health Department [] is totally aware” but abortion remains illegal “[b]ecause if anything goes wrong, they could prosecute … it's a taboo situation” (Pheterson and Azize, 2008).

7. No designated place for abortion services.

Abortion stigma also manifests in the ways in which physical spaces are or are not designated in health facilities. The procedure is marginalized and not provided with the same resources as other reproductive health procedures as a result of institutional or structural stigma. This emerged in three of the included studies (Freedman et al., 2010; Freeman and Coast, 2019; Ireland et al., 2020). In one study, some health care workers felt that this example of structural stigma toward the procedure led individuals to leave the hospital and perhaps seek clandestine services. A lack of awareness of the standards and guidelines for the delivery of abortion service within institutions also inhibited availability (Freedman et al., 2010).

## 4 Discussion

Previous research has alluded to the relationship between abortion stigma and quality in abortion care. Existing literature supports a definition of quality in abortion care that includes a range of features of healthcare and of the patient experience (Dennis et al., 2016; Darney et al., 2018). Reviews have also demonstrated how particular elements of quality, such as patient-centred care, are impacted by stigma, and can affect a person's overall experience (Altshuler and Whaley, 2018; Dennis et al., 2016). Stigma-related fears, including those related to condemnation and mistreatment, often lead abortion seekers to self-manage their abortions (Moseson et al., 2020). Furthermore, avoiding disclosure is regularly discussed as a mechanism for avoiding stigma (Astbury-Ward et al., 2012), making abortion seekers' expectations and experiences of treatment and provider-client relationships an important part of quality in abortion care (Baum et al., 2021; Georgsson et al., 2019; Doran and Nancarrow, 2015). Similarly, integration of abortion care into overall obstetrics and gynecology services is challenging due to the associated stigma (Freedman et al., 2010) and has important consequences for the professional lives of providers (Martin et al., 2014). A systematic review of barriers and facilitators to first-trimester abortion services in low- and middle-income countries highlighted staff harassment, insufficient hospital resources and costs as challenges to timely, respectful, and quality care (Doran and Nancarrow, 2015). However, to our knowledge the two phenomena have not previously been explicitly linked and clearly articulated.

**Fig. 2:**
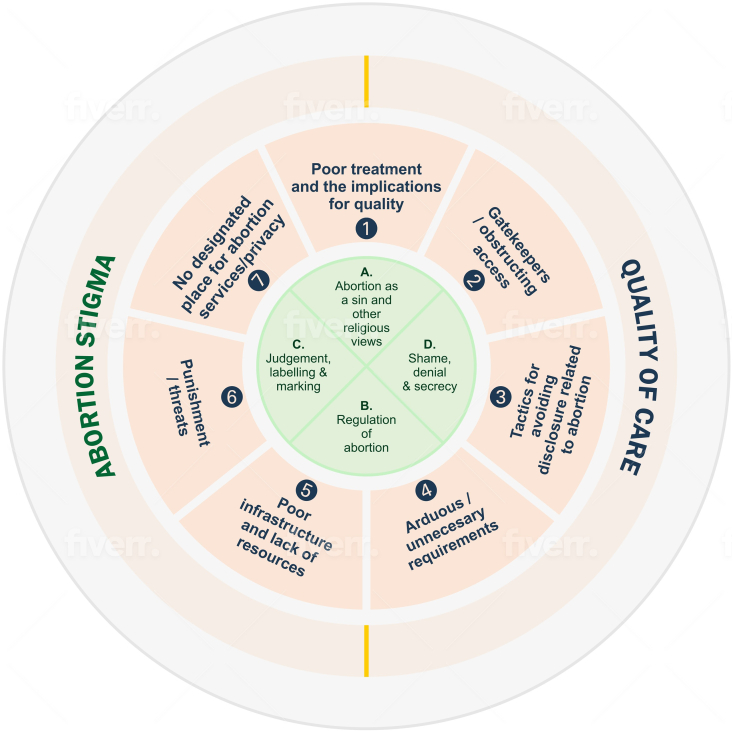
*The intersection of abortion stigma and quality in abortion care*.

In this scoping review, by intentionally applying the interface of two existing frameworks to the qualitative evidence and simultaneously allowing for the emergence of new data, we have captured the specific ways in which stigma operates to inhibit quality in abortion care due to these and additional factors at all levels of the abortion stigma ecological model, beyond the client-provider interaction (Fig. 2). All papers discussed here describe instances of abortion stigma that had implications for at least one of the six features of the WHO framework on quality of health care. This evidence complements the abortion stigma-adapted WHO quality of care framework suggested by the inroads network by illustrating how the postulated stigma-related barriers to quality abortion care occur in practice. Our inductive approach to the analysis strengthens and advances the inroads framework and thinking on the relationship between abortion stigma and quality in abortion care by basing it squarely in the published evidence. Furthermore, this evidence indicates specific areas for development of abortion stigma reduction interventions to improve quality in abortion care.

Existing research highlights various factors such as structure (health care system infrastructure, laws, and policies), process (technical competence, client-provider interactions, decision making, information provision, ancillary services, and support), output (procedures provided) and outcomes (client and community knowledge and attitudes, demographic trends, and morbidity and mortality) as critical for evaluation of health services (Dennis et al., 2016). This review, however, focuses in on concrete areas ripe for intervention. For example, training interventions designed to improve client-provider interactions could include values exploration exercises (Turner et al., 2018) to address gatekeeping applied by providers in determining who qualifies for care or those who refuse care. Similar efforts have proven successful when tackling cultural, social, or religious norms as well (Cockrill et al., 2013), all factors highlighted in this review. To appreciate the way in which health system infrastructure impacts care seeking, evidence from this review suggests the need to evaluate informal policies applied by providers, programme managers and institutions.

Although not explicitly included in our analysis, the legal and policy environment have important implications for abortion stigma and safety in abortion care (Ostrach, 2016). Restrictive legal environments can increase the stigmatization of providers and people accessing services and lead to unsafe abortion (Ostrach, 2016; Turan and Budhwani, 2021). As such, higher level interventions focused on policy makers are also needed to ensure that all levels of stigma are being addressed. Such interventions might include advocacy efforts focused on universal health coverage, for example, and be tailored to suit the context.

As demonstrated in this review, existing literature also points to the impacts associated with cost of care. Coping strategies (Ilboudo et al., 2015) and economic deterioration due to, among other factors, loss of assets, incurred debt, and loss of productivity, can have dire consequences for individuals and communities at large (Ilboudo et al., 2015; Sundaram et al., 2013). However, additional, and informal fees applied at the point of care (Duggal, 2004) not only have economic implications, but have a direct impact on quality in abortion care as exemplified here. Inclusion of abortion-related care would not only address the immediate concerns of individual abortion seekers, but also legitimize abortion as part of reproductive health care.

Abortion stigma and resultant care, loss of status, and discrimination is a violation of human rights (United Nations Human Rights, Office of the High Commissioner, 2020). States have an obligation to undertake measures to prevent and eliminate discrimination, stigmatization and negative stereotyping related to abortion care (Human Rights Council, 2018; Committee on the Elimination of Discrimination against Women, 2016). Barriers that lead women to resort to unsafe abortion must be eliminated (Human Rights Council, 2018; Committee on the Elimination of Discrimination against Women, 2016) and this includes ensuring that women are not prevented from accessing health services by health professionals’ exercise of conscientious objection (Committee on Economic, 2016; Committee on the Elimination of Discrimination against Women, 1999).

There are limitations to this scoping review. In the process of locating relevant qualitative literature, as we relied entirely on the database searches, some studies may have been missed. We also acknowledge that, though two researchers reviewed and analysed the evidence, our judgments and assessments of the literature could be considered subjective. However, the studies included represent a variety of geographical contexts, and the use of qualitative data ensures that the participant's experience is centred in the analysis.

Some argue that abortion stigma has become far too all encompassing, obscuring the specificities of the theory, and limiting its use as a tool for health and social justice (Kumar, 2013). Furthermore, using abortion stigma as the organizing principle for research, as we have here, has been criticised in the literature (Millar, 2020). It is argued that such framing of discourse may reinforce the negative concepts about abortion that abortion stigma research is aiming to diminish. However, existing research suggests that while abortion stigma is not the only determinant of unsafe abortion or poor quality in abortion care, nor may it be the primary determinant, it matters because it is a significant moderator of quality in abortion care. In fact, the evidence in this review suggests that quality in abortion care cannot be fully assessed without the consideration of the role of abortion stigma. Furthermore, overlooking the implications of abortion stigma for quality in abortion care may result in deleterious outcomes for the health and wellbeing of people seeking abortion. As such, it deserves attention and integration into frameworks used to assess the conditions of abortion health care services.

Further research should quantify the presence and magnitude of these manifestations in the quantitative literature and contribute to the development of indicators of quality in abortion care that include measures of abortion stigma, both as an outcome and to monitor practices, and the development of abortion stigma reduction interventions to improve quality in abortion care.

## Figures and Tables

**Table:** 

	#	Searches	Results
**Abortion stigma**		TI “stigma” OR AB “stigma” OR TI “stigma, social” OR AB “stigma, social” OR TI “shame” OR AB “shame” OR TI “guilt” OR AB “guilt” OR TI “discrimination” OR AB “discrimination” OR TI “prejudice” OR AB “prejudice” OR TI “negative” OR AB “negative” OR TI “judg*ment” OR AB “judg*ment” OR TI “stereotype” OR AB “stereotype” OR TI “blame” OR AB “blame” OR TI “secre*” OR AB “secre*” OR TI “disclos*” OR AB “disclos*” OR “isolation” OR AB “isolaton” OR TI “moral*” OR AB “moral*” OR TI “harass*” OR AB “harass*” OR TI “insult*” OR AB “insult*”	
**Abortion care**		(MH “abortion, induced+)” OR TI “abortion” OR TI “induced abortion” OR TI “induced abortions” TI “legal abortion” OR TI “legal abortions” OR AB “abortion” OR AB “induced abortion” OR AB “induced abortions” AB “legal abortion” OR AB “legal abortions” OR (TI “pregnancy” AND “termination) OR TI “pregnancy termination” OR TI “unintended pregnancies” OR TI “unintended pregnancy” OR TI “unwanted pregnancy” OR TI “unwanted pregnancies” OR (AB “pregnancy” AND “termination) OR AB “pregnancy termination” OR AB “unintended pregnancies” OR AB “unintended pregnancy” OR AB “unwanted pregnancies” OR (TI “unsafe”) AND (TI “abortion, induced” OR TI “abortion”) AND (TI “induced” OR TI “induced abortion” OR TI “abortion”) OR (AB “unsafe”) AND (AB “abortion, induced” OR AB “abortion”) AND (AB “induced” OR AB “induced abortion” OR AB “abortion”) OR TI “aborto” OR AB “aborto” OR TI “menstruation” OR (TI “menstrual” AND TI “regulation) OR AB “menstruation” OR (AB “menstrual” AND AB “regulation) OR (TI “delayed” AND TI “menstruation”) OR (AB “delayed” AND AB “menstruation”) OR (TI “suspended” AND TI “menstruation”) OR (AB “suspended” AND AB “menstruation”) OR (TI “cleaning” AND TI “uterus”) OR (TI “uterine” AND TI “cleaning” OR (AB “cleaning” AND AB “uterus”) OR (AB “uterine” AND AB “cleaning”) OR (TI “post” AND TI “abortion” AND TI “contraception”) OR (AB “post” AND AB “abortion” AND AB “contraception”)	
**Qualitative methods**		TI “life experiences” OR AB “life experiences” OR TI “human science” OR AB “human science” OR TI “discourse* analysis” OR AB “discourse* analysis” OR TI “narrative analysis” OR AB “narrative analysis” OR TI “lived experience*” OR AB “lived experience*” OR TI “field research” OR AB “field research” TI “field stud*” OR AB “field stud*” OR TI “giorgi*” OR AB “giorgi*” OR TI “husserl* OR AB “husserl*” OR TI “merleau ponty*” OR AB “merleau ponty*” OR TI “van kaam*” OR AB “van kaam*” OR TI “van manen*” OR AB “van manen*” OR TI “spiegelberg*” OR AB “spiegelberg*” OR TI “colaizzi*” OR AB “colaizzi*” OR TI “heidegger*” OR AB “heidegger*” OR TI “participant observ*” OR AB ““participant observ*” OR TI “data saturat*” OR AB “data saturat*” OR TI “semiotics” OR AB “semiotics” OR TI “heuristic” OR AB “heuristic” OR TI “hermeneutic*” OR AB “hermeneutic*” OR TI “etic” OR AB “etic” OR TI “emic” AB “emic” OR TI “focus group*” OR AB “focus group*” OR TI “purpos*” OR AB “purpos*” OR TI “sampl*” OR AB “sampl*” OR TI “constant compar*” OR AB “constant compar*” OR TI “grounded research” OR AB “grounded research” OR TI “grounded stud*” OR AB “grounded stud*” OR TI “grounded theor*” OR AB “grounded theor*” OR TI “phenomenol*” OR AB “phenomenol*” OR TI “ethnon*” OR AB “ethnon*” OR TI “qualitative” OR AB “qualitative” OR TI “ethnological research” OR AB “ethnological research” OR TI “ethnography” OR AB “ethnography” OR TI “phenomenology” OR AB “phenomenology” OR TI “discourse analysis” OR AB “discourse analysis” OR TI “theoretical sample” OR AB “theoretical sample” OR TI “thematic analysis” OR AB “thematic analysis” OR TI “content analysis” OR AB “content analysis” OR TI “observational method*" OR AB “observational method*" or TI “purposive sample” OR AB “purposive sample” OR TI “qualitative validity” OR AB “qualitative validity” OR TI “grounded theory” OR AB “grounded theory” OR TI “action research” OR AB “action research” OR TI “naturalistic inquiry” OR AB “naturalistic inquiry” OR TI “interview*" OR AB “interview*" OR TI “narratives” OR AB “narratives” OR TI “videorecording” OR AB “videorecording” OR TI “audiorecording” OR AB “audiorecording” OR TI “historical record*" OR AB “historical record*"	

**Table:** 

SECTION	ITEM	PRISMA-ScR CHECKLIST ITEM	REPORTED ON PAGE #
TITLE			
Title	1	Identify the report as a scoping review.	1
**ABSTRACT**			
Structured summary	2	Provide a structured summary that includes (as applicable): background, objectives, eligibility criteria, sources of evidence, charting methods, results, and conclusions that relate to the review questions and objectives.	4
**INTRODUCTION**			
Rationale	3	Describe the rationale for the review in the context of what is already known. Explain why the review questions/objectives lend themselves to a scoping review approach.	5
Objectives	4	Provide an explicit statement of the questions and objectives being addressed with reference to their key elements (e.g., population or participants, concepts, and context) or other relevant key elements used to conceptualize the review questions and/or objectives.	5
**METHODS**			
Protocol and registration	5	Indicate whether a review protocol exists; state if and where it can be accessed (e.g., a Web address); and if available, provide registration information, including the registration number.	6
Eligibility criteria	6	Specify characteristics of the sources of evidence used as eligibility criteria (e.g., years considered, language, and publication status), and provide a rationale.	6
Information sources^1^	7	Describe all information sources in the search (e.g., databases with dates of coverage and contact with authors to identify additional sources), as well as the date the most recent search was executed.	6
Search	8	Present the full electronic search strategy for at least 1 database, including any limits used, such that it could be repeated.	26
Selection of sources of evidence^2^	9	State the process for selecting sources of evidence (i.e., screening and eligibility) included in the scoping review.	6
Data charting process^3^	10	Describe the methods of charting data from the included sources of evidence (e.g., calibrated forms or forms that have been tested by the team before their use, and whether data charting was done independently or in duplicate) and any processes for obtaining and confirming data from investigators.	6
Data items	11	List and define all variables for which data were sought and any assumptions and simplifications made.	6 and 25
Critical appraisal of individual sources of evidence^4^	12	If done, provide a rationale for conducting a critical appraisal of included sources of evidence; describe the methods used and how this information was used in any data synthesis (if appropriate).	N/A
Synthesis of results	13	Describe the methods of handling and summarizing the data that were charted.	6
**RESULTS**			
Selection of sources of evidence	14	Give numbers of sources of evidence screened, assessed for eligibility, and included in the review, with reasons for exclusions at each stage, ideally using a flow diagram.	17
Characteristics of sources of evidence	15	For each source of evidence, present characteristics for which data were charted and provide the citations.	19
Critical appraisal within sources of evidence	16	If done, present data on critical appraisal of included sources of evidence (see item 12).	N/A
Results of individual sources of evidence	17	For each included source of evidence, present the relevant data that were charted that relate to the review questions and objectives.	19
Synthesis of results	18	Summarize and/or present the charting results as they relate to the review questions and objectives.	6
**DISCUSSION**			
Summary of evidence	19	Summarize the main results (including an overview of concepts, themes, and types of evidence available), link to the review questions and objectives, and consider the relevance to key groups.	12
Limitations	20	Discuss the limitations of the scoping review process.	13
Conclusions	21	Provide a general interpretation of the results with respect to the review questions and objectives, as well as potential implications and/or next steps.	13
**FUNDING**			
Funding	22	Describe sources of funding for the included sources of evidence, as well as sources of funding for the scoping review. Describe the role of the funders of the scoping review.	1

*JBI = Joanna Briggs Institute; PRISMA-ScR = Preferred Reporting Items for Systematic reviews and Meta-Analyses extension for Scoping Reviews.*

*From: Tricco AC, Lillie E, Zarin W, O'Brien KK, Colquhoun H, Levac D et al. PRISMA Extension for Scoping Reviews (PRISMAScR): Checklist and Explanation. Ann Intern Med. 2018; 169:467–473.*https://doi.org/10.7326/M18-0850*.*

1 Where *sources of evidence* (see second footnote) are compiled from, such as bibliographic databases, social media platforms, and Web sites.

2 A more inclusive/heterogeneous term used to account for the different types of evidence or data sources (e.g., quantitative and/or qualitative research, expert opinion, and policy documents) that may be eligible in a scoping review as opposed to only studies. This is not to be confused with *information sources* (see first footnote).

3 The frameworks by Arksey and O'Malley (6) and Levac and colleagues (7) and the JBI guidance (4, 5) refer to the process of data extraction in a scoping review as data charting.

4 The process of systematically examining research evidence to assess its validity, results, and relevance before using it to inform a decision. This term is used for items 12 and 19 instead of “risk of bias” (which is more applicable to systematic reviews of interventions) to include and acknowledge the various sources of evidence that may be used in a scoping review (e.g., quantitative and/or qualitative research, expert opinion, and policy document).

## Data Availability

Data will be made available on request.
